# Cancer Chemopreventive Ability of Conjugated Linolenic Acids

**DOI:** 10.3390/ijms12117495

**Published:** 2011-11-02

**Authors:** Takuji Tanaka, Masashi Hosokawa, Yumiko Yasui, Rikako Ishigamori, Kazuo Miyashita

**Affiliations:** 1 The Tohkai Cytopathology Institute: Cancer Research and Prevention (TCI-CaRP), 5-1-2 Minami-uzura, Gifu 500-8285, Japan; 2 Faculty of Fisheries Sciences, Hokkaido University, 3-1-1 Minato-cho, Hakodate, Hokkaido 041-8611, Japan; E-Mail: hoso@fish.hokudai.ac.jp; 3 School of Veterinary Medicine, Rakuno Gakuen University, 582 Midorimachi, Bunkyodai, Ebetsu, Hokkaido 069-8501, Japan; E-Mail: y-yasui@rakuno.ac.jp; 4 Division of Cancer Development System, Carcinogenesis Research Group, National Cancer Research Institute, Chuo-ku, Tokyo 104-0045, Japan; E-Mail: rishigam@ncc.go.jp; 5 Faculty of Fisheries Sciences, Hokkaido University, 3-1-1 Minato-cho, Hakodate, Hokkaido 041-8611, Japan; E-Mail: kmiya@fish.hokudai.ac.jp

**Keywords:** CLN, CLA, plant seed oils, cancer chemoprevention, PPARγ, p53

## Abstract

Conjugated fatty acids (CFA) have received increased interest because of their beneficial effects on human health, including preventing cancer development. Conjugated linoleic acids (CLA) are such CFA, and have been reviewed extensively for their multiple biological activities. In contrast to other types of CFAs including CLA that are found at low concentrations (less than 1%) in natural products, conjugated linolenic acids (CLN) are the only CFAs that occur in higher quantities in natural products. Some plant seeds contain a considerably high concentration of CLN (30 to 70 wt% lipid). Our research group has screened CLN from different plant seed oils to determine their cancer chemopreventive ability. This review describes the physiological functions of CLN isomers that occur in certain plant seeds. CLN are able to induce apoptosis through decrease of Bcl-2 protein in certain human cancer cell lines, increase expression of peroxisome proliferator-activated receptor (PPAR)-γ, and up-regulate gene expression of p53. Findings in our preclinical animal studies have indicated that feeding with CLN resulted in inhibition of colorectal tumorigenesis through modulation of apoptosis and expression of PPARγ and p53. In this review, we summarize chemopreventive efficacy of CLN against cancer development, especially colorectal cancer.

## 1. Introduction

Several fatty acids with conjugated double bonds are present in nature. These conjugated fatty acids (CFA) include conjugated dienes, trienes, and tetraenes. Examples are edible fats of ruminant origin, such as milk fat, tallow that contain conjugated linoleic acids (CLA) [1]. Many plant seed oils also contain conjugated trienoic fatty acids in the form of conjugated linolenic acids (CLN) [2,3]. In addition, aquatic plants including seaweeds contain conjugated polyenes in the forms of conjugated eicosapentaenoic acid (CEPA) and conjugated arachidonic acid (CAA) [4–6].

**Figure 1:**
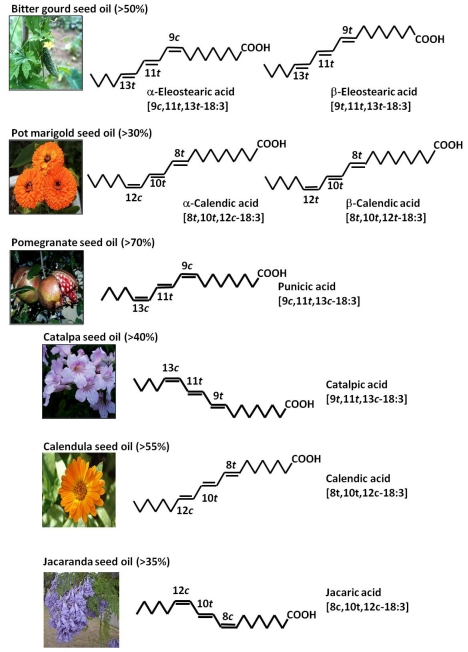
Conjugated linolenic acids (CLN) present in plant seed oils.

Most CFAs are 18-carbon compounds originating from oleic, linoleic, linolenic, and stearidonic acids. They occur in terrestrial plant lipids, especially seed oils, and include dienes, trienes, and tertraenes. The conjugated trienoic fatty acids from plant are mainly α-eleostearic acid (9c,11t,13t-18:3), catalpic acid (9t,11t-13c-18:3), punicic acid (9c,11t,13c-18:3), calendic acid (8t,10t,12c-18:3), and jacaric acid (8c,10t,12c-18:3) [7] (Figure 1). Calendic acid occurs in pot marigold seed oil, punicic acid in pomegranate seed oil, and α-eleostearic acid in tung and bitter gourd seed oils [8]. Main CLN of the plant seed oils are c,t,c, c,t,t, or t,t,c-isomers. Minor fatty acids, β-eleostearic acid (9t,11t,13t-18:3) and β-calendic acid (8t,10t,12t-18:3), are also present in some seed oils [8]. α-Eleostearic acid is the principal component of bitter gourd seed oil by contributing to more than 50% of oil; but the flesh of the bitter gourd contains catalpic acid [9].

A well-known conjugated diene and tetraene of plant origin is 10t 12t-18:2 [10] and α-parinaric acid (9c,11t,13t,15c-18:4) [11,12]. There are no reports on the occurrence of CFA with more than 18 carbon atoms in lipids of plant origin, and the major source of all these conjugated trienoic fatty acids may be seed oils. Most of seed oils contain positional and geometrical isomers of linolenic acid (18:3) with conjugated double bonds that is often referred to CLN. Unlike CLA that occurs at concentrations of less than 1% in nature, extremely high amounts of naturally occurring CLN is present in some seed oils. For example, 62.2% of pot marigold seed oil is calendic acid (8t,10t,12c-18:3), 83% of pomegranate seed oil is punicic acid (9c,11t,13c-18:3), 67.7% of tung seed oil and 56.2% of bitter gourd seed oil is α-eleostearic acid (9c,11t,13t-18:3), and 42.3% of catalpa see oil is catalpic acid (9t,11t,13c-18:3) [8]. Among seed oils that are sources of CLN, bitter gourd and pomegranate are edible plants, and catalpa is often used as Chinese medicine.

Although the bioactive properties of the CLA isomers have long been recognized [13,14], CLN have recently shown potent bioactivity. In a large number of *in vitro* and *in vivo* studies [15–18], CLN have displayed potent anti-inflammatory, immunomodulatory, anti-obese and anti-carcinogenic activities, along with the ability to improve biomarkers of cardio-vascular health. CLN isomers are naturally present in high concentrations in a large variety of seed oils and can also be produced *in vitro* by strains of lactobacilli and bifidobactena through the activity of the enzyme linoleic acid isomerase on α-linolenic acid [15,19]. In this review, we will summarize our findings in several studies showing possible cancer chemopreventive activities of CLN against colorectal cancer (CRC) and the mechanisms through which the activities are mediated.

## 2. CLN Inhibits Colon Carcinogenesis *in Vivo* Studies

About 30% of human cancers are considered to be associated with dietary habits and lifestyle. In particular, the amount and type of dietary fat influence development of certain types of cancer, such as colorectal malignancy [20–23]. CRC that is more common in developed countries is the third most commonly diagnosed cancer in the world [24]. In 2008, GLOBOCAN estimated that 1.23 million new cases of CRC were clinically diagnosed and killed more than 600,000 people [24]. It is well-known that CRC is linked to Western lifestyle, which often includes intake of high-fat diets, and the amount and type of dietary fat consumed are of particular importance for development of CRC [22,25–29]. Epidemiological studies suggest that high intake of fish and fish oil rich in *n*-3 polyunsaturated fatty acids (PUFA) correlates with a reduced risk of colorectal malignancy. Laboratory animal carcinogenesis studies indicate that *n*-3 PUFA are protective, whereas *n*-6 PUFA promote colorectal oncogenesis The mechanisms of protection by *n*-3 PUFA is mainly due to their interference with biosynthesis of two-series of prostaglandins (PGs) from arachidonic acid [30].

**Figure 2:**
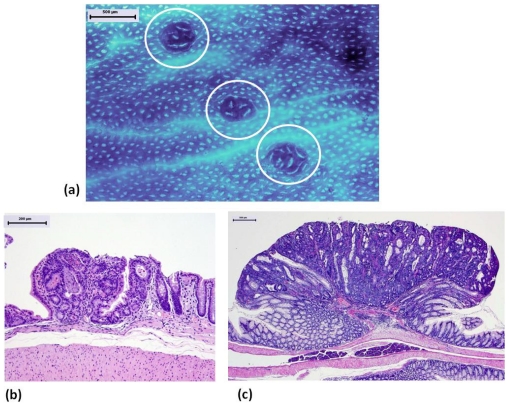
Colonic preneoplastic and neoplastic lesions induced by a colonic carcinogen, azoxymethane (AOM). (**a**) Aberrant crypt foci (ACF, circled) on colonic mucosa stained with methylene blue; (**b**) Tubular adenoma; (**c**) Tubular adenocarcinoma. ((**a**) Methylene blue stain; (**b**) and (**c**) Hematoxylin and eosin-stain; Bars: (**a**) (**c**) 500 m; (**b**) 200 m).

**Figure 3:**
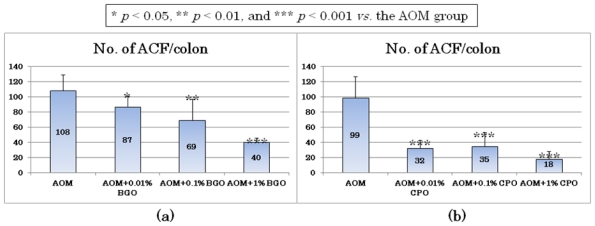
The numbers of aberrant crypt foci (ACF) induced by azoxymethane (AOM) from *in vivo* short-term assays determining the effects of 5-week feeding with (**a**) Bitter gourd (*Momordica charantia*) seed oil (BGO) and (**b**) Catalpa (*Catalpa ovata*) seed oil (CPO) in rats.

In a short-term animal study, dietary feeding with bitter gourd (*Momordica charantia*) seed oil (BGO) for 5 weeks at three dose levels (0.01, 0.1, and 1%) caused a significant reduction in the frequency of colonic aberrant crypt foci (ACF, Figure 2a), which are putative precursor lesions for CRC [31,32], in rats initiated with a colonic carcinogen, azoxymethane (AOM) [33]. A significant reduction in the multiplicity of ACF (Figure 3a) was found in rats fed the diet containing 0.01% BGO, in which 0.006% CLN (9c,11t,13t-18:3) was present. In another experiment, all three dose levels (0.01, 0.1, and 1%) of dietary administration with catalpa (*Catalpa ovata*) seed oil (CPO) that contains a large amount of catalpic acid (9t,11t,13c-18:3) for 5 weeks significantly inhibited ACF formation induced by AOM when compared to rats treated with AOM alone [34] (Figure 3b). Importantly, these studies showed that the diets containing BGO or CPO did not affect normal growth of colonic crypts and histology of liver, such as fatty liver. This may be explained by the findings in another *in vivo* study demonstrating that the BGO-containing diets significantly reduced free cholesterol levels with a trend toward an increase in high density lipoprotein, but did not affect the total cholesterol level [35]. Cell proliferation plays an important role in multistage carcinogenesis with multiple genetic changes. Dietary feeding with BGO or CPO lowered cell proliferation of ACF as well as normal appearing crypts of rats that received AOM, where the lesions and crypts were hyper-proliferation status due to injection of a colonic carcinogen, AOM [33,34]. Other natural compounds [36–38] and retinoids [39] that are potential cancer chmeopreventive agents possess similar effects [40–42]. As to the effects of BGO and CPO on apoptosis in the ACF, feeding with BGO or CPO increased apoptotic cells in ACF [33,34]. These findings were consistent with the anti-proliferative and apoptosis-inducing effects of CLN found *in vitro* studies with human colon cancer cell lines [43–45].

**Figure 4:**
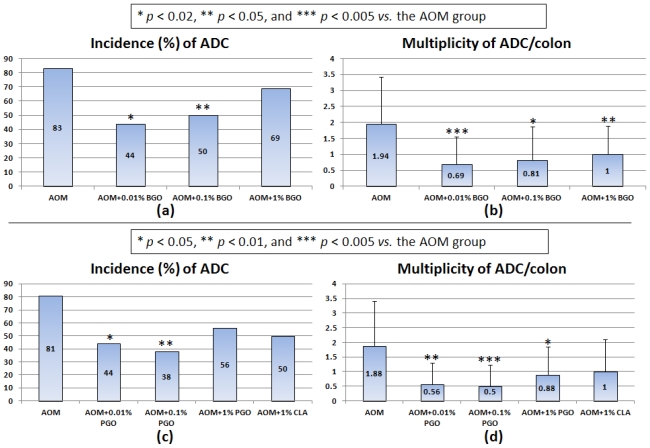
The incidences and multiplicities of adenocarcinoma (ADC) induced by azoxymethane (AOM) *in vivo* long-term assays determining the effects of 32-week feeding with (**a**) (**b**) BGO and (**c**) (**d**) PGO in rats. (**a**) (**c**) the incidences of ADC; and (**b**) (**d**) the multiplicities of ADC.

In a subsequent long-term *in vivo* assay, we confirmed chemopreventive ability of BGO against CRC development in rats [46]. Dietary administration of BGO rich in CLN (9c,11t,13t-18:3) for 32 weeks significantly lowered the incidence of colonic adenoma (Figure 2b) and adenocarcinoma (Figure 2c) induced by AOM in rats without causing any adverse effects (Figure 4a). The multiplicities (number of adenocarcinoma per rat) of CRC were also reduced when fed the BGO-containing diets at all three dose levels (0.01, 0.1, and 1%) (Figure 4b). Pomegranate (*Punica granatum* L.) seed oil (PGO) rich in other CLN isomer (9c,11t,13c-18:3) that was given to rats in their diet for 32 weeks also inhibited AOM-induced colorectal carcinogenesis, as estimated by the occurrence of colorectal adenocarcinoma (Figure 4c,d) [47]. Although the protective effects of BGO and PGO against CRC development were not dose-dependent, dietary feeding with BGO and PGO during the post-initiation stage suppressed progression of adenoma to malignant epithelial neoplasm (adenocarcinoma) [46,47]. In the long-term *in vivo* studies, we found interesting findings that dietary feeding with BGO or PGO increased expression of peroxisome proliferator-activated receptor (PPAR)-γ expression in the colonic mucosa [46,47]. PPARs that are members of the nuclear receptor superfamily have three different isoforms: PPARα, PPARδ, and PPARγ PPARs are ligand-activated transcription factors, and they are implicated in tumor progression, differentiation, and apoptosis. PPAR ligands are reported to activate PPAR signaling and exert cancer prevention and treatment *in vitro* and/or *in vivo* studies [48–50]. The findings are consistent with that *in vitro* studies using human CRC cell lines [44,45], where CLN (9c,11t,13t-18:3) was able to induce apoptosis in human colon cancer cells and enhance PPARγ expression in the CRC cells. Lipid peroxidation may be another possible explanation for the inhibitory effects of BGO and PGO on colorectal carcinogenesis [51]. Human CRC cells, DLD-1, were inoculated into nude mice, and then they were gavaged with CLA (9c,11t- and 10t,12c-18:2) and CLN (9c,11t,13t-18:3). The CLA and CLN treatments resulted in retardation of tumor growth, and the inhibitory effects of tumor growth was strong in the order of CLN >9c,11t-CLA > 10t,12c-CLA. CLN treatment caused DNA fragmentation and increased lipid peroxidation in tumor cells. Similar findings were observed in other study on the effects of CEPA [52], where CEPA had extremely strong anti-tumor effects on transplanted tumor cells, when compared to CEPA and CLA. In the tumor cells inoculated into the mice, the membranous phospholipid hydroperoxide and thiobarbituric acid reactive substances (TBARS) levels increased, when mice were given CEPA, suggesting the involvement of lipid peroxidation in the anti-carcinogenic effects of CEPA.

*In vivo* experimental animal studies demonstrating that dietary feeding with BGO [33,46], PGO [47], and CPO [34] exerts their chemopreventive activities in rat colorectal carcinogenesis, CLA isomers (9c,11t-18:2 for BGO and PGO; and 9t, 11t-18:2 for CPO) were detected in the liver lipids of rats fed with CLN [35,53]. CLA is a powerful anti-carcinogenesis agent in the rat mammary carcinogenesis with an effective range of 0.1 to 1.0% in the diet [54–56]. Therefore, the potential cancer chemopreventive effects of CLN are considered to be partly due to the presence of CLA isomers derived from CLN. A possible pathway for the formation of CLA in the liver lipids of rats fed BGO, PGO, and CPO is the bioconversion of CLN (9c,11t,13t-18:3, 9c,11t,13c-18:3, and 9t,11t,13c-18:3) to CLA (9c,11t-18:2 and 9t,11t-18:2) [35,53], but this bioconversion has not been confirmed in human. Although this may contribute to the inhibitory effects of CLN on colorectal carcinogenesis, other factors such as direct action of CLN as PPARγ ligand [46,47] and acceleration of lipid peroxidation followed by apoptosis [51] should be taken into account because powerful cancer chemopreventive activity of CLN at low dose levels was observed in our colorectal carcinogenesis studies [33,46,47]. Also, other mechanisms, including anti-inflammatory action, are considered for the inhibitory effects of CLN on colorectal carcinogenesis [32,48,57].

## 3. Growth-Inhibitory Effects of CLN *in Vitro* Studies

Naturally occurring CLN inhibit growth of variety of cancer cells and difference in activity was observed among different CLN isomers. Calendic acid (8t,10t,12c-18:3) from pot marigold did not affect the growth of SV-T2, SV40-transformed mouse fibroblasts, but α-eleostearic acid (9c,11t,13t-18:3), catalpic acid (91,11t,13c-18:3), and punicic acid (9c,11t,13c-18:3) were cytotoxic to the cells [58]. Similar anti-growth effects of α-eleostearic, catalpic, and punicic acids were observed in case of human monocytic leukemia cell line, U-937 [58]. Difference in cis/trans configuration among the CLN isomers did not influence their cytotoxicic ability. Also, the growth inhibitory effects of each 9,11,13-CLN isomer with either c-t-c, c-t-t or t-t-c configuration was greatly enhanced by adding an antioxidant, butylated hydroxytoluene to fatty acid [58]. This suggests involvement of lipid peroxidation in the cytotoxic effects of 9,11,13-CLN on human cancer cells. The oxidative stabilities of three types of 9,11,13-18:3 isomers were comparable, but was lower than the 8t,10t,12c-18:3 isomer [58]. Therefore, the cytotoxity of 9,11,13-18:3 that was greater than 8.10,12-18:3 may be partly due to their different susceptibilities to peroxidation [58].

The cytotoxic and anti-growth effects of CLN were also observed other human cancer cell lines, DLD-1 (colorectal adenocarcinoma), HepG2 (well-differentiated hepatocellular carcinoma), A549 (lung alveolar cell carcinoma), and HL-60 (acute promyelocytic leukemia) [51]. A fatty acid mixture rich in CLN (α-elcostcaric acid; 9c,11t,13t-18:3) showed dose-dependent growth-inhibitory effects via activation of the apoptotic pathway. The effects on a human CRC cell line (DLD-1) were stronger than two CLA isomers, 9c,11t-18:2 and 10t,12c-18:2. In the assays, the apoptotic promoting factors, caspases were activated by adding of α-eleostearic acid-rich fatty acid mixture to DLD-1 cells. The treatment with the fatty acid mixture resulted in an increase of amounts of membrane phospholipid peroxidation in DLD-1 cells, as measured by the TBARS values. In contrast, addition of α-tocopherol suppressed oxidative stress and apoptosis that were induced by α-eleostearic acid-rich fatty acid mixture.

The mechanisms underlying the cytotoxicity and apoptosis induced by α-eleostearic acid in Caco-2 cells (human colorectal adenocarcinoma) is suspected to be modulation of lipid peroxidation. However, the cytotoxic effects of β-eleostearic acid and β-calendic acid, which have all-trans-conjugated double bonds, were not completely reduced in the presence of α-tocopherol. This may suggest the presence of pathway(s) other than lipid peroxidation in the reduction of cell viability of Caco-2 cells by β-eleostearic and β-calendic acids. On the other hand, the cytotoxic effects of β-eleostearic acid on DLD-1 cells were not observed when α-tocopherol was added. Therefore, the metabolic and signal transduction systems in Caco-2 cells may contribute to different anti-cancer and anti-growth effects among CLN isomers, suggesting that further investigations are required for a better understanding of the specific mechanisms underlying the cytotoxic and apoptosis-inducing effects of β-eleostearic and β-calendic acids.

Major CLN present in the plant seed oils are c,t,c, c,t,t, or t,t,c-isomers. In addition, β-eleostearic acid (9t,11t,13t-18:3) and β-calendic acid (8t,10t,12t-18:3) are also present in some seed oils as minor fatty acids [8]. These all-trans-CLN isomers are contained as mixtures of CLN that are chemosynthesized by alkaline isomerization of linolenic acid [59]. Growth inhibition effects and induction of DNA fragmentation by β-eleostearic acid and β-calendic acids in Caco-2 cells were greater than α-eleostearic and α-calendic acids with cis configuration [43]. Furthermore, down-regulation of an anti-apoptotic protein bcl-2 mRNA and up-regulation of a pro-apoptotic protein bax mRNA in Caco-2 cells by β-eleostearic acid were greater than that by α-eleostearic acid. These findings indicate that the configuration of conjugated double bonds is important in cytotoxicity and apoptosis induced by CLN in Caco-2 cells and all-trans-CLN isomers act as more effective tumor-growth inhibitory compounds. When compared to CLA isomers with cis configuration, the greater tumor-inhibitory and apoptosis-inducing effects by all trans isomer of CLA on human CRC cell lines were demonstrated in a report by Beppu *et al.* [60]. In their study, three different human CRC cell lines, Caco-2, HT-29, and DLD-1, were incubated with 9c,11c-18:2, 9c,11t-18:2, 10t,12c-18:2 or 9t,11t-18:2, the strongest effect was observed in the treatment with 9t,11t-18:2, followed by 10t,12c-18:2, 9c,11c-18:2, and 9c,11t-18:2.

## 4. Molecular Mechanisms by Which CLN Exert Anti-Carcinogenesis and Anti-Tumor Growth Effects

The anti-carcinogenic effects of CLN and CLA have further been confirmed by Yasui *et al*. [45]. In their study, free fatty acids prepared from BGO (BGO-FFA) containing more than 60% α-eleostearic acid (9c,11t,l3t-l8:3) exhibited strong tumor-growth inhibition and apoptosis induction in three human CRC cell lines, DLD-1, HT-29, and Caco-2, the effects being greater than CLA (9c,11t-18:2). The study also demonstrated that the inhibitory effects of CLN were associated with modulation of PPAR-γ expression, which is one of the target molecules for suppressing development of cancer and other chronic diseases [48–50,61,62]. The treatment with BGO-FFA in Caco-2 cells resulted in approximately 3-fold increase in the expression of PPARγ protein in a dose-dependent manner, which was greater than an increase (1.5-fold) by troglitazone [45]. Cancer-retardant activity of CLA has also been reported to be associated with its ability to activate PPARγ [63]. PPARγ is predominantly found in normal adipose tissue [64], but is also expressed in cancer cells developed in a variety of tissues, including colorectum [65,66], breast [67], prostate [68], and tongue [69]. PPARγ activation induces growth arrest and apoptosis in CRC [70,71] and breast cancer cell lines [72]. In experimental colorectal carcinogenesis, a specific PPARγ ligand, troglitazone, effectively suppresses the development of ACF initiated with AOM and promoted with dextran sodium sulfate in rats [50,73]. PPARγ ligands, such as troglitazone and 15-deoxy-delta 12,14-prostaglandin J2 (15-d-PGJ2), cause growth-inhibition and induction of apoptosis in human cancer cell lines [71,72,74].

In another study by Yasui *et al*., the effects (reduction of cell viability and apoptosis induction) of CLN (9c,11t,13t-18:3) on the growth of HT-29 cells were much greater than that found in Caco-2 cells [44]. In general, expression of the PPARγ protein in HT-29 and DLD-1 cells is higher than that of Caco-2 cells. Therefore, 9c,11t,13t-18:3 as well as troglitazone arc more effective in growth inhibition and apoptosis induction of CRC cells with highly expressed PPARγ protein. HT-29, Caco-2, and DLD-1 cells are poorly differentiated, well-differentiated moderately differentiated adenocarcinomas, respectively [75,76]. Combination treatments with irinotecan and 5-fluorouracil resulted in different anti-cancer effect among CRC cell lines [77]. The differential responses of 9c,11t,13t-18:3 and troglitazone against HT-29, Caco-2 and DLD-1 cell lines may be depend on differences in cell differentiation of adenocarcinoma cells and PPARγ expression level.

The p53, growth-arrest, and GADD45 play important roles in the pathways of growth-inhibition and apoptosis induction in a variety of cancer cells [78,79]. Interestingly, the up-regulation of growth arrest and of DNA damage-inducible gene, GADD45, was observed in Caco-2 cells that received BGO-FFA [45]. Up-regulation of GADD45 mRNA in the Caco-2 cells treated with BGO-FFA was induced in a dose-dependent manner [45]. Up-regulation of p53 mRNA was also observed in Caco-2 cells treated with BGO-FFA [45]. Takekawa and Saito [80] identified GADD45 as a critical mediator of apoptosis triggered by the activation of JNK and/or p38, via MTK1/MEKK4 MAPK signaling pathways. GADD45 is also a target gene induced by the p53 tumor suppressor [81]. Although down-stream signaling from GADD45 that is affected by BGO-FFA is still unknown, we suspected that up-regulation of GADD45 and p53 contributes to apoptosis induction in Caco-2 cells treated with BGO-FFA.

## 5. Conclusion and Future Perspectives

CFA, such as CLA, CLN, and other polyenoic fatty acids, are indisputably reported to be novel biomolecules possessing potential health benefits, but it generally only occurs in very low quantities (<1%) in products of natural origin. The fact that high amounts of naturally occurring CLN are present in certain plant seed oils suggests it is much more accessible and easily available for dietary use than previously thought. CLN is thus a CFA with potential use as a functional food component in nutraceuticals. However, increased interest in CLN as a potential biomolecule and multi-biological function should be corroborated with information on its oxidative stability. In conclusion, CLN has a great potential as an ingredient in functional and health food formulations. Further, the exact biological effects CLN on the pathophysiological systems must be established in respect to its oxidative stability, since CLN is more susceptible to oxidation than corresponding non-conjugated fatty acid, α-linolenie acid [58]. Also, safety of CLN in humans [82–84] should be confirmed if CLN is to be used as a nutraceutical.
